# Identification of *moa*A3 gene in patient isolates of *Mycobacterium tuberculosis *in Kerala, which is absent in *M. tuberculosis *H37Rv and H37Ra

**DOI:** 10.1186/1471-2334-5-81

**Published:** 2005-10-04

**Authors:** Suma Sarojini, Smitha Soman, Indulakshmi Radhakrishnan, Sathish Mundayoor

**Affiliations:** 1 Mycobacterial Research Group, Rajiv Gandhi Centre for Biotechnology, Thiruvananthapuram, Kerala, India

## Abstract

**Background:**

Tuberculosis is endemic to developing countries like India. Though the whole genome sequences of the type strain *M. tuberculosis *H37Rv and the clinical strain *M. tuberculosis *CDC1551 are available, the clinical isolates from India have not been studied extensively at the genome level. This study was carried out in order to have a better understanding of isolates from Kerala, a state in southern India.

**Results:**

A PCR based strategy was followed making use of the deletion region primers to understand the genome level differences between the type strain H37Rv and the clinical isolates of *M. tuberculosis *from Kerala. PCR analysis of patient isolates using RD1 region primers revealed the amplification of a 386 bp region, in addition to the expected 652 bp amplicon. Southern hybridization of genomic DNA with the 386 bp amplicon confirmed the presence of this new region in a majority of the patient isolates from Kerala. Sequence comparison of this amplicon showed close homology with the *moa*A3 gene of *M. bovis*. In *M. bovis *this gene is present in the RvD5 region, an IS*6110 *mediated deletion that is absent in *M. tuberculosis *H37Rv.

**Conclusion:**

This study demonstrates the presence of *moa*A3 gene, that is absent in *M. tuberculosis *H37Rv and H37Ra, in a large number of local isolates. Whether the *moa*A3 gene provides any specific advantage to the field isolates of the pathogen is unclear. Field strains from Kerala have fewer IS*6110 *sequences and therefore are likely to have fewer IS*6110 *dependent rearrangements. But as deletions and insertions account for much of the genomic diversity of *M. tuberculosis*, the mechanisms of formation of sequence polymorphisms in the local isolates should be further examined. These results suggest that studies should focus on strains from endemic areas to understand the complexities of this pathogen.

## Background

Tuberculosis remains one of the most life threatening diseases and has been declared as a global emergency in 1993 by the World Health Organization (WHO) [1]. Failure in adhering to the strict drug regimen has led to the emergence of multi drug resistant isolates of this pathogen. This, combined with the problem of HIV, worsens the TB menace in developing countries. However, the main reason for the failure to eradicate tuberculosis lies in the biological properties of the infecting organism and its ability to persist in a latent state in the macrophages.

Bacterial strains within a single species exhibit variations in their properties such as pathogenicity, host specificity, virulence, adaptation to particular habitats and drug resistance. The passaging of *M. tuberculosis *H37Rv and *M. bovis *BCG for several decades outside the human host have induced changes in the genome of the pathogen and have also altered their virulence characteristics. Whole genome sequences of the type strain *M. tuberculosis *H37Rv [2], the clinical strain *M. tuberculosis *CDC1551 [3] and *M. bovis *[4] are already available. Many researchers have used a number of comparative analysis techniques like subtractive hybridization and microarray to identify differences in the genomes of laboratory strains and vaccine strains. Three genomic regions which are absent in *M. bovis *BCG but present in *M. bovis *and *M. tuberculosis *were first described using subtractive hybridization [5]. DNA microarray based studies between H37Rv and BCG have shown that 16 RDs (Regions of Differences) are deleted in BCG [6]. Similarly, whole genome comparison studies have shown six deletion regions in *M. tuberculosis *H37Rv – RvD1 to RvD5 and TbD1 [7].

Our earlier studies on the clinical isolates from Kerala on IS*6110 *polymorphism have shown that a large number of isolates have few or no copy of the sequence [8]. This made about fifty percent of strains untypable using IS*6110*. These results have been corroborated by studies from other endemic areas in India as well as outside [9-12]. This has prompted us to speculate on whether there are major differences in the genome in the field strains from endemic areas. Therefore, we examined these strains to see the distribution of the different RD regions. Here we report the presence of a genomic region in the clinical isolates of *M. tuberculosis*, which is absent in the type strains H37Rv and H37Ra.

## Methods

### Mycobacterial strains and DNA isolation

The type strains used for the study included *Mycobacterium tuberculosis *H37Rv, H37Ra and *M. bovis *BCG. These were grown in Middlebrook 7H9 Broth (Difco Laboratories) supplemented with OADC enrichment (Difco Laboratories) and 0.05% glycerol (USB Corporation). Field strains of *Mycobacterium tuberculosis *were those isolated from sputum samples of tuberculosis patients from different parts of Kerala. The strains were biochemically tested for Catalase, Niacin and Nitrate for identification. They were characterised by IS*6110 *fingerprinting. Drug resistance pattern was also studied using the four major frontline drugs *viz*, isoniazid, rifampicin, ethambutol and streptomycin.

DNA was isolated from cells pelleted from liquid culture using glass beads in a minibead beater. The DNA was precipitated after phenol:chloroform extraction using 3M Sodium acetate (pH 5.2) and 100% ethanol and dissolved in TE buffer, pH 8.0.

### PCR amplification of RD1 region

Oligonucleotide primer pairs used for the study were RD1DLa: 5'-AGA TGA AGA CCG ATG CCG CTA C -3' and RD1DRa: 5'-CCC GTG TTT CGC TAT TCT ACG C-3'. PCR was performed in a final volume of 30 μl using 1.25 units of Taq DNA Polymerase (Promega Corporation) for each reaction. After initial denaturation, amplification was done using a PCR thermal cycler (BioRad) for 35 cycles of 94°C/40 sec, 64°C/1 min, 72°C/1 min followed by a final extension of 72°C/7 min. To identify the flanking sequences of the 386 bp region, another set of primers were used (moaFP: 5'-CCCATCGTGGTCGTTCACC-3' and moaRP: 5'-CGATGGCAGCGGTTTACAG-3') which was expected to amplify a 1254 bp product.

### Southern hybridization

Genomic DNA from *M. tuberculosis *H37Rv, H37Ra, *M. bovis *BCG and the clinical isolates digested using *Eco*R I (New England Biolabs) was separated on agarose gels, transferred to nylon membrane (Hybond) and probed with α^32^P [dCTP] labelled PCR product. After overnight hybridization at 65°C, the blot was washed with increasing stringency of SSC-SDS and exposed to an activated Phosphor screen (Kodak). The screen was then scanned using Personal Molecular Imager FX (BioRad) and the picture was visualized using the software Quantity One (BioRad).

### Cloning and sequencing

PCR products separated on agarose gel were eluted using GFX ™ PCR DNA and Gel Band Purification kit (Amersham Pharmacia Biotech Inc). The eluted DNA was cloned into pGEMT Easy vector (Promega Corporation). Plasmid DNA for sequencing was purified using Nucleospin Plasmid kit (Macherey-Nagel) in accordance with manufacturers' instructions. Plasmids were digested with *Eco*R I to check for the presence of inserts. DNA sequencing by cycle sequencing method with the fluorescent dye terminator (Big Dye Terminator Cycle Sequencing Ready Reaction Kit, (PE Biosystems)) was carried out with T7 and SP6 promoter primers in an automated sequencer (ABI Prism 310).

## Results

### Screening of RD1 by PCR

**Figure 1:**
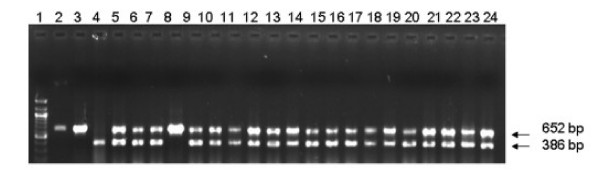
**PCR of clinical isolates of *M. tuberculosis *for RD1 region. **Lane 1: 100 bp marker, lane 2: H37Rv, lane 3: H37Ra, lane 4: *M. bovis *BCG, lanes 5–24: Clinical isolates RGTB 29, 37, 40, 43, 55, 60, 70, 86, 87, 93, 95, 109, 110, 123, 142, 144, 154, 167, 177, 193 respectively.

**Figure 2:**
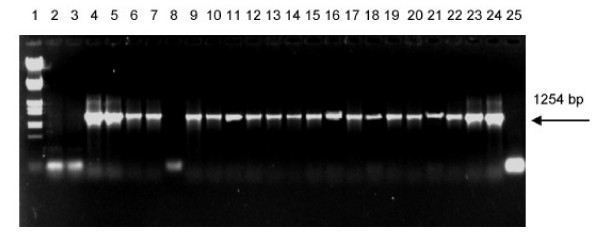
**PCR of clinical isolates of *M. tuberculosis *for *moa*A3 gene. **Lane 1: Marker- λ DNA double digest (*Eco*R I/*Hind *III), lane 2: H37Rv, lane 3: H37Ra, lane 4: *M. bovis *BCG, lanes 5–24: Clinical isolates RGTB 29, 37, 40, 43, 55, 60, 70, 86, 87, 93, 95, 109, 110, 123, 142, 144, 154, 167, 177, 193 respectively, lane 25: Negative Control.

RD1, the most significant region of difference between *M. tuberculosis *and *M. bovis *BCG is a 9505 bp long region absent in all the different BCG substrains. PCR primers were designed to amplify regions within RD1 to find out polymorphism between type strains and the clinical isolates. PCR using RD1DLa and RD1DRa primers was expected to amplify a 652 bp fragment (comprising of Rv3874 and Rv3875, coding for *cfp*10 and *esat *6) in *M. tuberculosis *H37Rv and the clinical isolates. In H37Rv and H37Ra the expected 652 bp band was observed. In BCG the 652 bp band was absent as expected, but a 386 bp fragment was amplified. The clinical isolates showed both 652 and 386 bp fragments. A set of twenty patient isolates from Kerala was used for the initial screening. Of these, only one isolate (RGTB43), did not have the 386 bp amplicon. (Fig. 1). Later we screened a total of one hundred isolates from Kerala by PCR and all except three showed the presence of the 386bp amplicon (Data not shown). A second PCR using primes designed from the surrounding regions of *moa*A3 gene was done to confirm the presence of the full ORF in clinical isolates. The expected amplicon of 1254 bp was obtained in all those clinical isolates which had the 386 bp fragment (Fig 2).

### Southern blot

**Figure 3:**
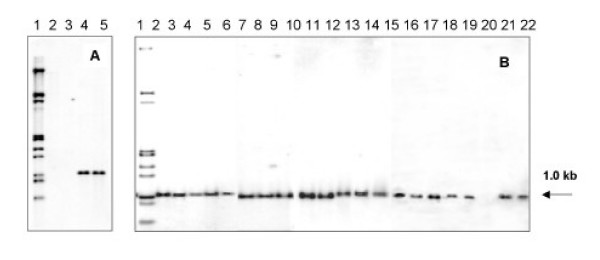
**Southern hybridization of *Eco*R I digested genomic DNA probed with radiolabelled 386 bp PCR product. **Panel A- Lane1: Marker – λ DNA double digest (*Eco*R I/*Hind *III) lane2: H37Rv, lane 3: H37Ra, lane 4: *M. bovis *BCG, lane 5: pool of DNA from local isolates. Panel B – Southern hybridization to DNA from individual isolates. Lane 1: Marker – λ DNA double digest (*Eco*R I/*Hind *III), lane 2: *M. bovis *BCG, Lanes 3–22: Isolates RGTB 70, 86, 87, 93, 95, 109, 110, 123, 142, 144, 154, 167, 177, 193, 29, 37, 40, 43, 55, 60 respectively.

To confirm the results of the PCR, *Eco*R I digested genomic DNA from *M. bovis *BCG, *M. tuberculosis *H37Rv and H37Ra and pooled DNA from all the 20 clinical isolates (called local pool) were subjected to Southern hybridization using radioactively labelled 386 bp fragment from *M. bovis *BCG. Local pool and *M. bovis *BCG showed a signal corresponding to about 1.0 kb (Fig. 3A) while H37Rv and H37Ra were negative. Southern hybridization of each of the individual clinical isolate was then carried out for confirming the result and all, except RGTB 43, showed a positive signal (Fig. 3B). The strain RGTB 43 was negative by PCR as well.

### DNA sequence homology

**Figure 4:**
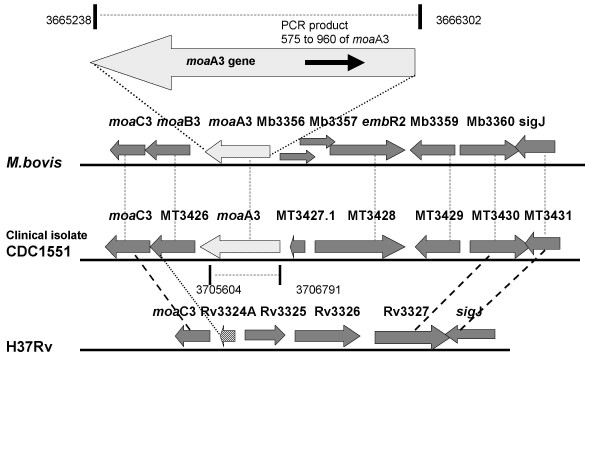
**A diagrammatic representation of *moa*A3 and the surrounding regions. **Comparison of the region comprising *moa*A3 gene and the surrounding genes in *M. bovis*, H37Rv and the clinical isolate, CDC1551. All data from references [19, 20, 21, 22]. The coamplified PCR product is shown as a thick line in the *moa*A3 locus. The amplicon extends from 575 to 960 of *moa*A3 gene in *M. bovis*. The genome coordinates of the *moa*A3 gene in *M. bovis *and *M. tuberculosis *CDC1551 are shown. The thin dotted lines indicate corresponding genes in *M. bovis *and CDC1551. The bold dotted lines indicate similar genes in CDC1551 and H37Rv. Rv3324A is differentially shown since it's a truncated gene and has only partial nucleotide similarity to CDC1551 *moa*B3 gene.

Sequencing of the 386 bp amplicon cloned into pGEMT Easy vector was carried out using T7 and SP6 promoter primers. The sequence data obtained was compared to the whole genome of *M. bovis *[19]*M. tuberculosis *[20] and 100% sequence homology was obtained with *M. bovis *whereas *M. tuberculosis *H37Rv showed only 61%. The upstream and downstream sequences of this 386 bp region were identified by searching the *M. bovis *genome database. It was found that the sequenced fragment did not belong to the RD1 region. Instead, it was found to be part of RvD5, a deletion in the type strain H37Rv (Fig. 4). This region corresponds to *moa*A3 gene in *M. bovis *which codes for molybdenum cofactor biosynthesis protein A, MoaA1.

The *moa*A3 gene was present in 97 of the 100 clinical isolates tested (details not presented). These isolates had varying IS*6110 *and drug resistance profiles suggesting the possible absence of a relationship between the *moa*A3 fragment, IS*6110 *copy number and drug resistance profile.

## Discussion

Studies using subtractive hybridization [5] and microarrays [6] have identified 16 regions, (ranging in size from 2–12.7 kb), in *M. tuberculosis *H37Rv which are absent in *M. bovis *BCG. Deletions are also reported in H37Rv – RvD1 to RvD5 and TbD1 [7]. These results suggest that generation of deletions may be a major mechanism for creating genetic diversity among the members of the complex. On this basis, we sought to screen the clinical isolates of *M. tuberculosis *from Kerala for differences in the RD regions. Initially, we used primers spanning RD1 region, since RD1 is the most important region of difference and is deleted in all the substrains of *M. bovis *BCG [6]. The loss of RD1 is one major genetic event that contributes to the attenuation of BCG, and its reintroduction into an attenuated strain resulted in a significant increase in virulence [13].

Of the nine open reading frames predicted within the 9.5 kb RD1 region, ORFs coding for *cfp*10 and *esat6 *are considered to be very important as there is vigorous host response to these proteins. Amplification using primers that span this region was expected to give a 652 bp PCR product. But the PCR results revealed an extra amplicon of 386 bp in the local isolates and BCG. Further characterization by sequencing and homology search indicated that this region is a part of the *moa*A3 gene which codes for molybdopterin cofactor protein A in *M. bovis*. The sequence of the 386 bp amplicon obtained from the local strains showed 100% homology with *M. bovis *as compared to 61% with *M. tuberculosis *H37Rv. The PCR primers that we made spanning the RD1 region was similar to portions of the *moa*A3 gene in the RvD5 region, which resulted in the amplification of the 386 bp fragment. This amplicon spanned the nucleotides 575 to 960 of the *moa*A3 gene in *M. bovis *(Fig 4). The *moa*A3 gene is absent in H37Rv, but another gene in the biosynthetic pathway, *moa*C3, was the closest to the 386 bp amplicon, with a homology of 61%. Database searches revealed that the *moa*A3 gene is present in the CDC1551 in the RvD5 region as well. To confirm the location of the *moa*A3 gene in our isolates, a second PCR designed to amplify the flanking sequences of *moa*A3 gene was performed. The results confirmed the location of the *moa*A3 gene in the clinical isolates from Kerala. In *M. bovis *(Mb3355) the gene is 1065 bp long while in CDC 1551, the *moa*A3 gene (MT3427) is 1189 bp long, due to an additional 123 bp in the C terminal region. In the overlapping region, CDC1551 has 100% homology with *M. bovis moa*A3. Genome comparison studies have shown that *moa*A3 is one among the few genes that is present in CDC1551 and absent in H37Rv [3]. Southern hybridization studies done in our lab confirmed that *moa*A3 gene is absent in the type strains *M. tuberculosis *H37Rv and H37Ra and is present in most of the clinical isolates in Kerala as well as in *M. bovis *BCG. Since *moa*A3 gene has been seen in the RvD5 region in both *M bovis *and in CDC1551, we have presumed that these genes are in the same region in our local strains as well, but these results need confirmation. The regions surrounding the *moa*A3 gene and the IS*6110 *elements flanking the RvD5 region in these local isolates merit further investigation.

Molybdopterin is a cofactor required for nitrate reductase and many other enzymes involved in anaerobic metabolism. Genes involved in the molybdopterin cofactor biosynthesis pathway are present in almost all organisms. *M. tuberculosis *H37Rv dedicates 21 genes to the biosynthesis of this cofactor [2]. But there is no gene homologous to the *moa*A3 found in *M. bovis*. This cofactor is thought to be involved in the biosynthesis of molybdopterin precurser Z from guanosine in *M. bovis*. Studies in *Escherichia coli *have suggested that these molybdoenzymes have the ability to hydroxylate or dehydroxylate certain compounds enabling the bacteria to detoxify them [14]. In addition, *E. coli *with defective *moa *show a decrease in the frequency of adaptive mutations [15]. Thus, one may infer that the *moa*A3 gene might have a role in the intracellular survival of the local *M. tuberculosis *strains or may provide some selective advantage to them. A recent study using a promoter trap vector has identified two of the genes, *moa*X and *moe*B1 as upregulated in mouse lungs upon infection [16]. A systematic study is required to understand the exact role of this protein in the lifecycle of this pathogen. At the same time, the effect due to the lack of *moa*A3 on *M. tuberculosis *H37Rv may be difficult to quantify as the remaining array of *moa *genes could be expected to complement any lost activity.

The RvD5 region from which the amplicon was generated is an IS*6110 *mediated deletion in the type strain H37Rv [7]. IS*6110*, a powerful genetic marker for strain differentiation [17] has also been shown to play an important role in mediating genomic rearrangements and deletions in mycobacteria. In fact, four of the five genomic deletions in *M. tuberculosis *H37Rv (except RvD1) are predicted to be IS*6110 *mediated recombinations [7]. But IS*6110 *mediated alterations may not provide much selective advantage to the bacteria from endemic areas such as Kerala, as a large percentage of the isolates have very few copies of IS*6110 *[8]. Insertions and deletions are important in the evolution of a bacterial species. *M. tuberculosis*, considered an evolutionarily "young" pathogen, would not be expected to have undergone extensive variations in its genome [18]. But, in spite of this, differences could be detected between the laboratory strains and clinical isolates, both by sequence analysis as in the case of CDC1551 [3] and by PCR as in this study. In a scenario of few/no copies of IS*6110 *other insertion sequences or mobile genetic elements could be involved in these variations. Therefore, a detailed study of the genome of field strains from different endemic regions would provide more insights into the diversity of this pathogen.

## Conclusion

This study, demonstrates the presence of the *moa*A3 gene in a large number of local isolates. This gene has been shown to be present in *M. bovis*, but not in H37Rv or H37Ra. The results obtained suggest that the population of strains in endemic areas is different from type strains, as suggested earlier by our analysis of IS*6110*. The field strains may also vary between different endemic regions. So the strains from endemic areas need to be examined in greater detail to understand the complexities of this pathogen. Such an understanding is essential for us to be able to plan adequate control measures for tackling what is appearing to be the world's number one killer.

## Abbreviations

RD: Region of Difference

RGTB: Rajiv Gandhi Centre for Biotechnology Tuberculosis isolates

PCR: Polymerase Chain Reaction

## Competing interests

The author(s) declare that they have no competing interests.

## Authors' contributions

SS carried out most of the experiments, data analysis and wrote the manuscript. SS# did part of the experimental work. IR did the IS*6110 *work and contributed to the writing of the manuscript. SM conceived and co-designed the study, provided inputs for writing and supervised the study. All authors read and approved the final manuscript.

**Table 1:** Details of clinical isolates of *M. tuberculosis*. The resistance (R)/sensitivity(S) profile of the isolates to the four frontline anti- tuberculosis drugs and their IS*6110 *copy number are shown below.

RGTB No:	Drug resistance profile				IS*6110 *copy no:
		
	Isoniazid	Ethambutol	Rifampicin	Streptomycin	
29	R	R	S	S	1
37	S	S	S	S	1
40	S	R	S	S	0
43	S	R	S	S	9
55	R	R	S	S	1
60	R	R	S	R	2
70	S	S	S	S	1
86	R	R	R	S	1
87	R	R	S	S	3
93	R	S	S	S	1
95	S	S	S	S	6
109	S	S	S	S	12
110	R	S	S	S	8
123	S	S	S	S	0
142	R	R	S	R	10
144	S	S	S	S	1
154	R	S	R	S	1
167	S	R	S	S	14
177	S	R	S	S	1
193	R	R	R	S	1

## Pre-publication history

The pre-publication history for this paper can be accessed here:


